# Crystal structure of monkeypox H1 phosphatase, an antiviral drug target

**DOI:** 10.1093/procel/pwac051

**Published:** 2022-11-05

**Authors:** Wen Cui, Haojun Huang, Yinkai Duan, Zhi Luo, Haofeng Wang, Tenan Zhang, Henry C Nguyen, Wei Shen, Dan Su, Xi Li, Xiaoyun Ji, Haitao Yang, Wei Wang

**Affiliations:** Institute of Life Sciences, Chongqing Medical University, Chongqing 400016, China; Institute of Life Sciences, Chongqing Medical University, Chongqing 400016, China; Shanghai Institute for Advanced Immunochemical Studies and School of Life Science and Technology, ShanghaiTech University, Shanghai 201210, China; Institute of Life Sciences, Chongqing Medical University, Chongqing 400016, China; Shanghai Institute for Advanced Immunochemical Studies and School of Life Science and Technology, ShanghaiTech University, Shanghai 201210, China; Institute of Life Sciences, Chongqing Medical University, Chongqing 400016, China; Shanghai Institute for Advanced Immunochemical Studies and School of Life Science and Technology, ShanghaiTech University, Shanghai 201210, China; Shanghai Institute for Advanced Immunochemical Studies and School of Life Science and Technology, ShanghaiTech University, Shanghai 201210, China; Institute of Life Sciences, Chongqing Medical University, Chongqing 400016, China; State Key Laboratory of Biotherapy and Cancer Center, West China Hospital, Sichuan University and Collaborative Innovation Center of Biotherapy, Chengdu 610064, China; Institute of Life Sciences, Chongqing Medical University, Chongqing 400016, China; Institute of Life Sciences, Chongqing Medical University, Chongqing 400016, China; State Key Laboratory of Pharmaceutical Biotechnology, Department of Biotechnology and Pharmaceutical Sciences, School of Life Sciences, Nanjing University, Nanjing 210023, China; Shanghai Institute for Advanced Immunochemical Studies and School of Life Science and Technology, ShanghaiTech University, Shanghai 201210, China; Shanghai Clinical Research and Trial Center, Shanghai 201210, China; Tianjin International Joint Academy of Biotechnology and Medicine, Tianjin 300450, China; Institute of Life Sciences, Chongqing Medical University, Chongqing 400016, China

**Dear Editor,**

Since its appearance in May 2022, monkeypox has spread to >100 countries and afflicted tens of thousands of people. Individuals infected with monkeypox present a fever, an extensive characteristic rash, and usually swollen lymph nodes (Chatterjee et al., 2022). The number of confirmed cases worldwide continues to grow at a rapid rate, but the treatment to this highly infectious viral disease is still very limited. Identification of new targeted-therapies will be crucial to control of this emerging public health threat.

The monkeypox virus is an enveloped double-stranded DNA virus that belongs to the *Orthopoxvirus* genus of the *Poxviridae* family (Isidro et al., 2022). It has a very large genome (~200 kb) and codes around 200 proteins (Kugelman et al., 2014). Poxviruses express a dual specific phosphatase (H1) that de-phosphorylates signal transducer and activator of transcription 1 (STAT1) and blocks interferon signal transduction (Najarro et al., 2001; Mann et al., 2008). H1 is conserved in poxviruses and is essential for virus replication in cell culture (Liu et al., 1995). Inhibiting H1 expression results in greatly reduced infectivity. About 200 copies of H1 are packaged into newly formed viral particles and function in the early stage of viral infection (Liu et al., 1995). H1 has also been suggested to de-phosphorylate monkeypox proteins F18, A14, and A17 (Liu et al., 1995; Derrien et al., 1999; Traktman et al., 2000). Due to the importance of H1 in modulating interferon-signaling and viral replication, it serves as an attractive anti-poxvirus drug target.

To understand the mechanism for monkeypox H1 catalyzed dephosphorylation and provide an accurate structural model for drug discovery, we determined a crystal structure of H1 to 1.8 Å resolution (Fig. 1 and Table S1). Monkeypox H1 has 171 amino acid residues and the refined model includes residues 2–171 with well-fitting electron density. There is one H1 molecule in an asymmetric unit. Two H1 molecules are related by crystallographic symmetry and form a domain swapped dimer, which resembles a butterfly (Fig. 1A). The overall structure is composed of six α helices and four β strands. A four-stranded β-sheet is sandwiched by helices α2 and α3–α6 on either side. The active site is located near the C-terminus of the last β-strand. The two active sites are ~39 Å apart (Fig. 1A). There is a phosphate ion captured at each active site, representing the final stage of catalysis before the product is released.

**Figure 1.**
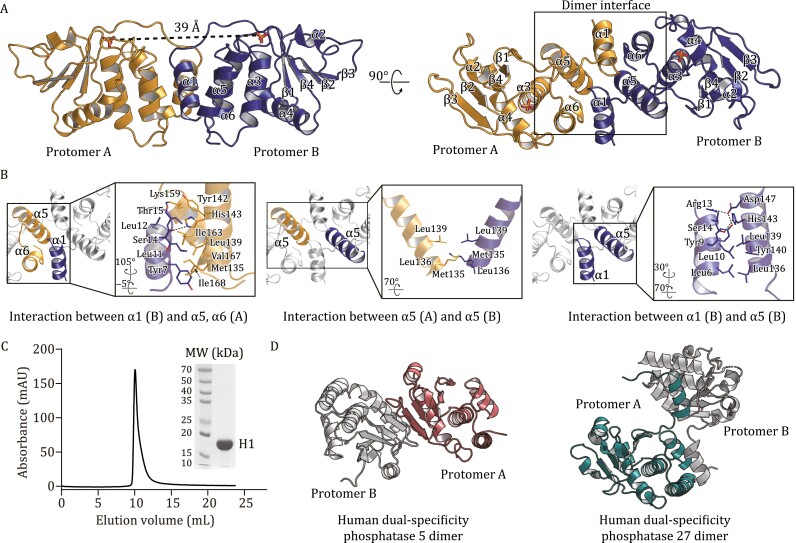
**Monkeypox H1 forms a domain swapped dimer.** (A) The overall structure of H1 dimer, shown in two different views. Two protomers are in yellow and blue, respectively. The secondary structure elements are labeled. The two active sites are on the same side of the dimer and ~39 Å apart. There is a phosphate ion in each active site. (B) Zoom in of the dimer interface, which consists three components. The residues that participate in dimerization are shown as stick models. Hydrogen bonds are shown as dashed lines. (C) Size exclusion chromatography of monkeypox virus H1. The curve shows H1 was eluted at 10 mL on a Superdex 75 increase 10/300 GL column (Cytiva, USA), which corresponds to a molecular weight of ~40 kDa. SDS-PAGE analysis of the purified H1 is shown on the right. (D) Dimeric strucutres of representative human phosphatases in the PTP/DSP family, namely, human dual-specificity phosphatase 5 (PDB ID: 2G6Z), and human dual-specificity phosphatase 27 (PDB ID: 2Y96). The protomer A of these structures are presented in the same angle as the corresponding protomer of monkeypox virus H1 in the right panel of Fig. 1A.

The N-terminal helix α1 from each protomer interchanges to mediate H1 dimerization. The α1 from one H1 protomer forms a four-helix bundle with three helices α4−α6 of the pairing protomer. The buried surface area between Protomer A and B is ~1000 Å^2^, which is stabilized by both hydrophilic and hydrophobic interactions. Residues Ser14 and Thr15 in α1 form hydrogen bonds with His143 and Tyr142/Lys159 of the other H1 protomer, respectively; whereas Tyr7, Leu11, and Leu12 participate in hydrophobic interactions with Met135, Leu139, Lys159, Ile163, Val167, and Ile168 from the pairing H1 molecule (Fig. 1B, left panel). In addition, three residues in α5, Met135, Leu136, and Leu139, face the symmetry related residues in the dimer to expand the hydrophobic interface (Fig. 1B, middle panel). α1 is also stabilized by intramolecular hydrogen bonds and hydrophobic interactions between residues of α1 and α5 (Fig. 1B, right panel). Size exclusion chromatography confirms the H1 dimer in solution, suggesting that dimerization represents its functional state (Fig. 1C).

The H1 active site consists of a Cys-Arg-Asp catalytic triad (Fig. 2A). The conserved Cys and Arg residues are in a loop between β4 and α4 (^109^HCVAGVNRS^117^), which is also known as the phosphate-binding loop. The arginine residue (Arg116) of this loop captures the phosphate ion, whose guanidinium group interacts with two phosphate oxygens through two hydrogen bonds with the distances of 2.9 and 3.0 Å, respectively (Fig. 2B). This important arginine residue guarantees efficient binding and orientation of the substrate. At the bottom of the catalytic pocket (Fig. 2B), the conserved Cys110 attacks the phosphorous atom during the de-phosphorylation reaction, resulting in a transient enzyme-phosphate intermediate. This intermediate is then hydrolyzed to generate inorganic phosphate and the regenerated enzyme. The sulfur atom of Cys110 is positioned in line with a phosphorous-oxygen (P-O) bond which corresponds to the one formed during the enzyme regeneration step (Fig. 2B). Asp79 is responsible for coordinating the water molecule, which is also hydrogen bonded to oxygen from the phosphate group (Fig. 2B). This residue functions as a general acid, facilitating both the formation of the enzyme-phosphate intermediate and its hydrolysis. Thus, the crystal structure represents the final step of catalysis before the product is released.

**Figure 2.**
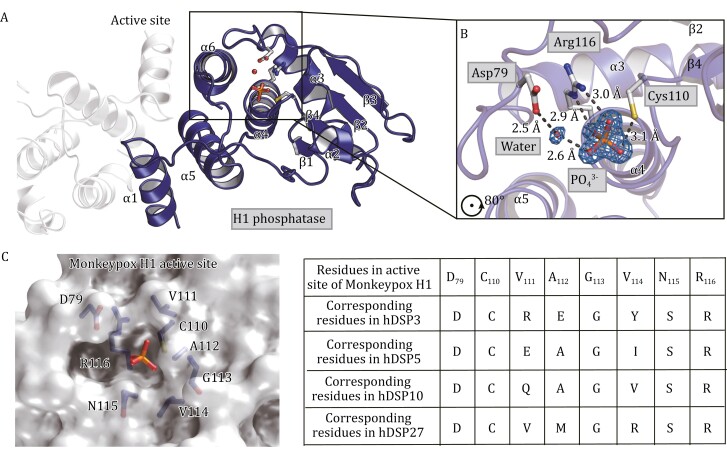
**The active center of H1, which captures the final step of catalysis before the product is release.** (A) The active site is located near the C-terminus of β4, which consists of a phosphate-binding loop (between β4 and α4) and a general acid loop (between β3 and α3). The Cys-Arg-Asp catalytic triad and the phosphate ion in active site are shown as stick models. Water is presented as a red sphere. (B) Zoom in of the active site. Hydrogen bonds are shown as dashed lines. The distances between the hydrogen bonded atoms are labeled. Simulated annealing omit map is shown for the phosphate ion and the water molecule. The map is generated with the standard composite omit map procedure implemented in Phenix (torsion angle simulated annealing with 5% of model omitted at a time). (C) The active site sequence is different among the phosphatases in the PTP/DSP family. The surface representation of the H1 active site is shown in the left panel, with the residues at its active site labeled. The residues at the active site of representative human phosphatases in the PTP/DSP family are shown in the right panel. hDSP3: human dual-specificity phosphatase 3; hDSP5: human dual-specificity phosphatase 5; hDSP10: human dual-specificity phosphatase 10. hDSP27: human dual-specificity phosphatase 27.

The high-resolution crystal structure of monkeypox H1 reveals at least two hot spots for drug discovery. The first hot spot is the dimer interface, which is unique among the members in the protein tyrosine phosphatase (PTP)/dual-specificity phosphatase (DSP) family (Fig. 1A and 1D). Blocking H1 dimerization may potentially inhibit its ability to dimerize and dephosphorylate the phosphor-tyrosine in activated STAT1, which is also a homodimer (Wenta et al., 2008; Jeong et al., 2014). In addition, the active site is another potential target for inhibition. Although the active sites of all phosphatases in PTP/DSP family are built around a phosphate-binding loop (with a sequence HCX_5_R(S/T)) and have a similar main chain structure, the side chains around the active center are different (Yuvaniyama et al., 1996; Tao and Tong, 2007; Jeong et al., 2014) (Fig. 2C), which affects their own substrate specificity and may allow the development of specific inhibitors.

In conclusion, we report a high-resolution crystal structure of the monkeypox H1 phosphatase that lays a solid foundation for its mechanistic study and the discovery of antiviral compounds against this emerging pathogen.

## Supplementary Material

pwac051_suppl_Supplementary_Material

## Footnotes

We are grateful to the staff at the BL18U1 at Shanghai Synchrotron Radiation Facility (SSRF, China), where data were collected. This work was supported by grants from the National Natural Science Foundation of China (Nos. 81902063, 81871639, and 92169109) and Ministry of Science and Technology of the People’s Republic of China (Nos. 2018YFA0507100 and 2020YFA0707500). This work was also supported by the Chongqing Science and Technology Commission (No. cstc2019jcyj-msxmX0135), Science and Technology Research Program of Chongqing Municipal Education Commission (No. KJQN202000442) and CQMU Chongqing Medical University (No. W0073) to W.W., and grants from Lingang Laboratory (No. LG202101-01-07), Science and Technology Commission of Shanghai Municipality (Nos. YDZX20213100001556 and 20XD1422900), Natural Science Foundation of Tianjin (No. 18JCJQJC48000) ,and Shanghai Municipal Development & Reform Commission Major Project (No. ZD2021CY001) to H.Y. This work was further supported by Shanghai Frontiers Science Center for Biomacromolecules and Precision Medicine, ShanghaiTech University.

H.Y. and W.W. conceived the project; W.C., H.Y., and W.W. designed the experiments; W.C., H.H., Z.L., and T.Z., cloned, expressed, purified, and crystallized proteins; Y.D. and H.W. collected the diffraction data; W.C., Y.D., H.W., and X.J. solved the crystal structure; H.H., W.C., Y.D., W.S., D.S., X.L., X.J., W.W., and H.Y. analyzed and discussed the data; H.C.N., X.J., W.W., and H.Y. wrote the manuscript.

Coordinates and structure factors for Monkeypox virus H1 has been deposited in Protein Data Bank (PDB) with accession number 8GZ4.

The authors have no conflicts of interest to declare that are relevant to the content of this article.
