# Supplementary material for: Crystal structure of monkeypox H1 phosphatase, an antiviral drug target
Source: Protein Cell. 2022 Nov 5;14(6):469–72. doi: 10.1093/procel/pwac051 (PMC10246720; doi:10.1093/procel/pwac051)
Supplement: pwac051_suppl_Supplementary_Material [file pwac051_suppl_supplementary_material.pdf]

Crystal structure of monkeypox H1 phosphatase, an antiviral drug target

Wen Cui[1,8], Haojun Huang[1,8], Yinkai Duan[2,8], Zhi Luo[1], Haofeng Wang[2], Tenan Zhang[1], Henry C. Nguyen[2], Wei Shen[3], Dan Su[1,4], Xi Li[1], Xiaoyun Ji[1,5,*], Haitao Yang[2,6,7,*], Wei Wang[1,*]

[1]Institute of Life Sciences, Chongqing Medical University, Chongqing, China

[2]Shanghai Institute for Advanced Immunochemical Studies and School of Life Science and Technology, ShanghaiTech University, Shanghai, China

[3]School of Life Science and Technology, ShanghaiTech University, Shanghai, China

[4]State Key Laboratory of Biotherapy and Cancer Center, West China Hospital, Sichuan University and Collaborative Innovation Center of Biotherapy, Chengdu, China,

[5]State Key Laboratory of Pharmaceutical Biotechnology, Department of Biotechnology and Pharmaceutical Sciences, School of Life Sciences, Nanjing University, Nanjing, China

[6]Shanghai Clinical Research and Trial Center, Shanghai, China

[7]Tianjin International Joint Academy of Biotechnology and Medicine, Tianjin, China

[8]These authors contributed equally: Wen Cui, Haojun Huang, Yinkai Duan.

*Corresponding author.

Correspondence: xiaoyun.ji@nju.edu.cn; yanght@shanghaitech.edu.cn; wangwei@cqmu.edu.cn

Keywords: crystal structure, dual specific phosphatase, monkeypox

Running title: Crystal structure of monkeypox H1 phosphatase

Contents

Materials and Methods

Supplementary Table 1

Materials and Methods

Cloning, protein expression and purification of monkeypox H1. The DNA coding for monkeypox H1 of the current monkeypox virus (MPXV) outbreak (isolate name MPXV_USA_2022_MA001; accession ON563414 in GenBank) was synthesized by Tsingke Biotech (China) and cloned into the pETDuet-1 expression vector using restriction sites *Eco*RI and *Nde*I, coding proteins with a N-terminal His-tag. The plasmid was verified by sequencing (Tsingke Biotech, China). H1 was expressed in Escherichia coli BL21(DE3) in Luria broth (LB) at 16 °C for 16-18 h with 0.5 mM IPTG. Bacteria expressing H1 were harvested and resuspended in a lysis buffer containing 20 mM Tris-HCl, pH 8.0, 0.5 M NaCl, 10 mM imidazole, 5 mM $MgCl_2$ and 10% glycerol and lysed by high pressure homogenization. After centrifugation (16,000 x g, 30 min at 4 °C), the supernatant was loaded onto a Ni-NTA column (GE Healthcare, USA). The column was washed using a buffer containing 20 mM Tris-HCl, pH 8.0, 0.2 M NaCl, 50 mM imidazole, 5 mM $MgCl_2$ and 10% glycerol and eluted using a similar buffer supplemented with 250 mM imidazole. The eluted protein was concentrated and purified by gel-filtration chromatography (Superdex 75 increase 10/300 GL, Cytiva, USA), using a buffer containing 20 mM Tris-HCl, pH 8.0, 500 mM NaCl and 5 mM $MgCl_2$.

Crystallization, data collection and structure determination. Crystals were grown by the sitting-drop vapor diffusion method. H1 was crystallized at 16 °C by mixing 1 μL protein (15 mg/mL) with 1 μL crystallization buffer containing 0.2 M Li_2SO_4, 0.1 M Bis-Tris pH 6.5 and 25% polyethylene glycol 3,350. The crystals were cryoprotected using the crystallization buffer supplied with 20% glycerol. X-ray diffraction data were collected on beamline BL18U1 at the Shanghai Synchrotron Radiation Facility at 100 K and at a wavelength of 0.97776 Å. Data integration and scaling were performed using XDS (Otwinowski and Minor, 1997). The structure was determined by molecular replacement method with the AutoSol program in PHENIX (Adams et al., 2010) using the structure of

Vaccinia Virus H1 (PDB: 3CM3) as a search model. The H1 model was initially built by the Autobuild program in PHENIX and subsequently subjected to iterative cycles of manual building in Coot (Emsley et al., 2010) and refinement in PHENIX.

Structural comparison with human phosphatases in the PTP/DSP family. The coordinates of monkeypox H1 was uploaded on DALI server (Holm, 2022) and search for proteins with similar structure in Protein Data Bank (using the PDB50 subset). 30 phosphatase structures were found to have a Z-score higher than 13.0, which indicates significant structural similarity. Among them, two human phosphatases are crystalized as dimer: human dual-specificity phosphatase 5 (hDSP5, PDB ID: 2G6Z) and human dual-specificity phosphatase 27 (hDSP27, PDB ID: 2Y96). Their dimerization mode was compared.

References

Adams, P.D., Afonine, P.V., Bunkóczi, G., Chen, V.B., Davis, I.W., Echols, N., Headd, J.J., Hung, L.W., Kapral, G.J., Grosse-Kunstleve, R.W., *et al.* (2010). PHENIX: a comprehensive Python-based system for macromolecular structure solution. Acta Crystallogr D Biol Crystallogr *66*, 213-221.

Emsley, P., Lohkamp, B., Scott, W.G., and Cowtan, K. (2010). Features and development of Coot. Acta Crystallogr D Biol Crystallogr *66*, 486-501.

Holm, L. (2022). Dali server: structural unification of protein families. Nucleic Acids Res *50*, W210-W215.

Otwinowski, Z., and Minor, W. (1997). Processing of X-ray diffraction data collected in oscillation mode. Methods Enzymol *276*, 307-326.

Supplementary Table 1. Data collection and refinement statistics

Property	Value
**Data collection**	
Space group	*I* 4 2 2
Cell dimensions	
a, b, c (Å)	62.48, 62.48, 170.494
α, β, γ (°)	90.00, 90.00, 90.00
Resolution (Å)	30.67-1.80 (1.86-1.80)
$I/\sigma I$	26.6 (4.4)
Redundancy	16.1 (16.7)
Completeness (%)	100.0 (100.0)
R_{merge}	7.4% (63.4%)
$CC_{1/2}$	99.9 (95.6)
**Refinement**	
Resolution (Å)	30.67-1.80 (1.83-1.80)
No. reflections	16132(1586)
R_{work} / R_{free}	0.172/0.196 (0.225/0.275)
No. atoms	
Protein	1385
Ligand/ion	5
Water	99
B-factors	
Protein	28.74
Ligand/ion	26.63
Water	36.90
R.m.s. deviations	
Bond lengths (Å)	0.008
Bond angles (°)	0.094

Statistics for the highest-resolution shell are shown in parentheses.
